# Effect of filgotinib, a selective JAK 1 inhibitor, with and without methotrexate in patients with rheumatoid arthritis: patient-reported outcomes

**DOI:** 10.1186/s13075-018-1541-z

**Published:** 2018-03-23

**Authors:** Mark Genovese, Rene Westhovens, Luc Meuleners, Annegret Van der Aa, Pille Harrison, Chantal Tasset, Arthur Kavanaugh

**Affiliations:** 1 Division of Immunology and Rheumatology, Stanford School of Medicine, Stanford, CA USA; 2 Department of Development and Regeneration KU Leuven, Skeletal Biology and Engineering Research Center; Rheumatology, University Hospitals Leuven, Leuven, Belgium; 3 Galapagos NV, Mechelen, Belgium; 4 University of California San Diego, La Jolla, CA USA; 5 Division of Immunology and Rheumatology, Stanford School of Medicine, 1000 Welch RD #203, Palo Alto, CA 94304 USA

**Keywords:** Rheumatoid Arthritis, Filgotinib, Patient-Reported Outcome Measures, JAK inhibitor

## Abstract

**Background:**

The aim was to assess patient-reported outcomes (PROs) in patients with rheumatoid arthritis (RA) treated with filgotinib during two phase 2b, 24-week, randomized, placebo-controlled studies.

**Methods:**

Patients with moderate-to-severe active RA and an inadequate response to methotrexate (MTX) were randomized to daily placebo or filgotinib 50 mg, 100 mg, or 200 mg as add-on therapy to MTX (NCT01888874) or as monotherapy (NCT01894516). At week 12, nonresponders receiving filgotinib 50 mg in both studies or placebo in the add-on study, and all patients receiving placebo as monotherapy, were re-assigned to filgotinib 100 mg. PROs were measured using the Health Assessment Questionnaire - Disability Index (HAQ-DI) including Patient Pain assessed by visual analog scale, and the Patient Global Assessment of Disease Activity (Patient Global), the Functional Assessment of Chronic Illness Therapy (FACIT)-Fatigue Scale (Version 4), and the 36-Item Short Form Health Survey (SF-36).

**Results:**

At week 12, improvements in all PROs, apart from the SF-36 mental component in the add-on study, were statistically better with filgotinib than placebo; some improvements were noted as early as the first assessment time point (week 1 or week 4). Filgotinib improved HAQ-DI by 0.58–0.84 points, FACIT-Fatigue by 6.9–11.4 points, Patient Global by 25.2–35.6 mm, and Pain by 24.2–37.9 mm; scores were maintained or improved to week 24. Across all PROs, more patients achieved minimal clinically important differences and normative values with filgotinib 200 mg than placebo. Patients re-assigned to filgotinib 100 mg at week 12 experienced improvements in PROs between weeks 12 to 24.

**Conclusions:**

Filgotinib as MTX add-on therapy or as monotherapy demonstrated rapid and sustained (to 24 weeks) improvements in health-related quality of life and functional status in patients with active RA.

**Trial registration:**

MTX add-on study: ClinicalTrials.gov, NCT01888874. Registered on 28 June 2013. Monotherapy study: ClinicalTrials.gov, NCT01894516. Registered on 10 July 2013.

**Electronic supplementary material:**

The online version of this article (10.1186/s13075-018-1541-z) contains supplementary material, which is available to authorized users.

## Background

Rheumatoid arthritis (RA) is a chronic, inflammatory disease, characterized by painful, swollen joints during periods of active inflammation [1]. With suboptimum management, joint damage may occur, leading to loss of function and disability, accompanied by systemic features such as anemia and weight loss during periods of disease activity [2, 3]. The clinical manifestations of RA challenge patients’ coping skills: quality of life (QoL) can become severely impaired both in terms of physical and mental health [3, 4]. Although therapeutic advances have been made over recent decades, patients still face serious challenges: side effects occur and treatment responses may be slow, incomplete, and unsustained.

Janus kinase (JAK) inhibitors offer promise as targeted synthetic disease-modifying antirheumatic drugs (tsDMARDs) by blocking JAK-mediated signaling pathways that are implicated in the pathology of RA [5–8]. Filgotinib (GLPG0634/GS-6034) is an oral, selective inhibitor of JAK1, based on both biochemical and whole-blood assays [9, 10].

Two 24-week, placebo-controlled, phase 2b studies demonstrated that filgotinib was well-tolerated and efficacious in treating the signs and symptoms of RA in patients with moderate-to-severe active RA and an inadequate response to methotrexate (MTX) [11, 12]. The DARWIN 1 study evaluated filgotinib as add-on therapy to MTX and the DARWIN 2 study assessed filgotinib as monotherapy. In both studies, significantly more patients achieved the primary endpoint, an American College of Rheumatology (ACR)20 response, after 12 weeks of treatment with filgotinib 200 mg or 100 mg (or with 50 mg in the monotherapy study) versus placebo, and responses were maintained or improved until the end of the 24-week treatment period. Importantly, ACR20 responses were achieved quickly – by 2 weeks for the higher doses [11, 12].

Patient-reported outcomes (PROs) provide insight into patient QoL and the impact of disease on functional capacity – they are important assessors of the value of new therapeutics for RA, alongside evaluations of clinical effects and safety. Both clinical studies measured PROs as prespecified secondary endpoints to ascertain the effect of filgotinib on health-related QoL (HRQoL). Here, we report in detail the findings from PROs measured in both the MTX add-on (DARWIN 1) and monotherapy (DARWIN 2) studies, including the speed of response, whether responses were clinically meaningful, the proportion of patients who achieved normalized PRO scores, and the influence of clinical response on PROs.

## Methods

Full methods have been published previously [11, 12]. In brief, both studies were 24-week, multicenter, randomized, double-blind, placebo-controlled, phase 2b, dose-finding studies of oral filgotinib.

### Study design and treatments

In the MTX add-on study (ClinicalTrials.gov identifier NCT01888874), filgotinib was administered orally in combination with a stable dose of MTX. Patients were randomized to receive placebo or one of three daily doses of filgotinib – 50 mg, 100 mg, or 200 mg – either once daily (q.d.) or as 25-mg, 50-mg, or 100-mg doses twice daily (b.i.d.), in a 1:1:1:1:1:1:1 ratio. In the monotherapy study (NCT01894516), patients were randomized to receive placebo or one of three filgotinib daily doses (50 mg, 100 mg, or 200 mg q.d.), in a 1:1:1:1 ratio. In both studies, randomization was stratified by geographical region and previous use of biologic DMARDs (bDMARDs) (to which < 10% of the total study population had been previously exposed).

At week 12, patients were assessed for response, defined as a 20% improvement in swollen joint count (SJC) based on 66 joints and tender joint count (TJC) based on 68 joints. Nonresponders in the placebo, 25 mg b.i.d., and 50 mg q.d. groups of the MTX add-on study were reassigned to the filgotinib 100 mg q.d. or 50-mg b.i.d. groups. In the monotherapy study, all patients in the placebo group and nonresponders in the filgotinib 50-mg q.d. group were reassigned to the filgotinib 100-mg q.d. group (Additional file 1: Figure S1). Responders continued in their allocated treatment group for the remainder of the study.

Written approval was obtained before initiation of the study from the Medical Ethical Committee of University Hospitals KU Leuven (reference ML9437; study site of the Principal Investigator for the DARWIN 1 study) and the University of California San Diego Human Research Protections Program (reference 140642; study site of the Principal Investigator for the DARWIN 2 study), and from the appropriate independent ethics committee or institutional review board for each of the 106 study centers in the DARWIN 1 and 59 study centers in the DARWIN 2 studies (Additional file 2: Document 1 and Additional file 3: Document 2, respectively). Both studies were conducted in accordance with the ethical principles of the Declaration of Helsinki, International Council on Harmonisation good clinical practice guidelines, and all applicable national and local laws and regulatory requirements. All patients provided written, informed consent prior to study participation.

### Patients

In both studies, enrolled patients were ≥ 18 years of age with a diagnosis of RA for ≥ 6 months prior to screening, met the 2010 ACR/European League Against Rheumatism criteria for RA and ACR functional classes I–III, had ≥ 6/66 SJC and ≥ 8/68 TJC and a screening serum C-reactive protein ≥ 0.7 times the upper limit of the laboratory normal range. Patients receiving oral glucocorticoids (≤ 10 mg/day) or nonsteroidal anti-inflammatory drugs had to be on a stable dose for ≥ 4 and ≥ 2 weeks, respectively, before baseline.

In the MTX add-on study, patients had been receiving MTX for ≥ 6 months and were on a stable dose (15–25 mg/week, oral or parenteral) 4 weeks before screening. In the monotherapy study, patients had shown an inadequate response to MTX (in the opinion of the treating physician) and were washed out from MTX at least 4 weeks prior to baseline.

### Outcomes and assessments

PROs were measured using the Health Assessment Questionnaire-Disability Index (HAQ-DI), including Patient Pain assessed on a visual analog scale (VAS) [13], Patient Global Assessment of Disease Activity (Patient Global) VAS [14], Functional Assessment of Chronic Illness Therapy (FACIT)-Fatigue Scale (Version 4), and 36-Item Short Form Health Survey (SF-36) [15, 16]. The three constituent PROs of the ACR response criteria, HAQ-DI, Patient Global and Patient Pain, were measured at baseline (day − 1) and at weeks 1, 2, 4, 8, 12, 16, 20, and 24. Two additional PRO measures, the FACIT-Fatigue Scale and SF-36, were measured at baseline and at weeks 4, 12, and 24. Changes from baseline in PRO parameters were examined, along with active group versus placebo comparisons. Minimal clinically important differences (MCIDs) were assessed as change from baseline, defined as a 0.22-point decrease on a scale of 0–3 for HAQ-DI scores [17], a 10-mm decrease on a scale of 0–100 mm for Patient Pain and Patient Global [18], a 4-point increase on a scale of 0–52 in FACIT-Fatigue score, [19] and a 2.5-point increase on a scale of 0–100 for SF-36 physical component summary (PCS) and mental component summary (MCS) scores [17]. At week 12, the proportions of patients who reached normalized PRO values were measured for HAQ-DI, FACIT-Fatigue, and PCS and MCS, based on normative values ≤0.5 for HAQ-DI (minimal disease activity) [20], ≥40 for FACIT-Fatigue [21], and ≥50 for PCS and MCS scores [22, 23]. The PRO instruments, MCIDs and normalized values used in this study are summarized in Additional file 4: Table S1.

### Sample sizes and statistical analyses

All randomized patients who received at least one dose of study drug were included in the intent-to-treat (ITT) population. Patients who switched treatments at week 12 were handled as discontinuations and data were imputed from week 12 onwards. Missing data were accounted for using a last observation carried forward computation. Between-group comparisons were done for each filgotinib dose group versus the placebo group. Hommel’s closed testing correction procedure was applied to adjust for multiplicity. Corrected *p* values <0.05 were considered indicative of a statistically significant comparison.

MCIDs and normative analyses were exploratory and were not tested for significance. Similarly, this study was not powered for any formal comparisons among the dose groups, or between the q.d. and b.i.d. regimens, which were examined in an exploratory fashion via a model containing both variables. No adjustment for multiplicity was made for these exploratory comparisons.

## Results

### Patient characteristics

A total of 594 patients in the MTX add-on study and 283 patients in the monotherapy study received at least one dose of study drug and were included in the ITT population. Additional file 1: Figure S1A and B illustrate patient flow through the studies.

Baseline patient demographics and disease characteristics by treatment group for both studies have been reported previously and were similar between the two overall study populations (Additional file 4: Table S2) and were balanced between the treatment groups within each study [11, 12].

Scores for PROs at baseline indicated a population with a high disease burden in line with moderate-to-severe RA activity scores; levels of disability reported were similar across the treatment groups in both studies (Table 1).

**Table 1:** Patient-reported outcomes at baseline (ITT populations)

	Methotrexate add-on study							Monotherapy study				
	Placebo (*N* = 86)	50 mg q.d. (*N* = 82)	100 mg q.d. *(N* = 85)	200 mg q.d. (*N* = 86)	25 mg b.i.d. (*N* = 86)	50 mg b.i.d. (*N* = 85)	100 mg b.i.d. (*N* = 84)	Placebo (*N* = 72)	50 mg q.d. (*N* = 72)	100 mg q.d. (N = 70)	200 mg q.d. (*N* = 69)	Score interpretation
HAQ-DI, mean (SE)	1.69 (0.06)	1.71 (0.07)	1.70 (0.07)	1.76 (0.06)	1.70 (0.05)	1.78 (0.06)	1.78 (0.07)	1.80 (0.06)	1.84 (0.07)	1.80 (0.07)	1.79 (0.06)	0–3; higher score indicates higher disability
Patient Global, mean (SE)	64.2 (1.96)	68.2 (2.23)	67.6 (2.09)	68.7 (2.09)	64.3 (1.95)	65.7 (1.92)	66.6 (2.20)	71.1 (2.02)	68.6 (2.41)	71.5 (2.23)	68.9 (2.07)	VAS score 0–100; higher score indicates lower health state
Patient Pain, mean (SE)	65.7 (2.16)	66.9 (2.20)	65.4 (2.41)	67.0 (2.16)	65.7 (2.23)	67.8 (2.12)	67.2 (2.19)	71.6 (2.37)	71.0 (2.38)	72.6 (1.85)	68.1 (2.35)	VAS score 0–100; higher score indicates more severe pain
FACIT-Fatigue, mean (SE)	26.2 (1.09)	26.2 (1.10)	26.6 (1.06)	25.2 (1.25)	28.1 (1.18)	26.2 (1.04)	25.6 (1.25)	25.1 (1.12)	25.1 (1.28)	24.8 (1.13)	24.8 (1.16)	0–52; higher score indicates better QoL
SF-36 PCS, mean (SE)	33.0 (0.71)	32.7 (0.75)	31.6 (0.79)	31.6 (0.64)	31.6 (0.69)	31.3 (0.73)	32.2 (0.78)	31.1 (0.70)	31.1 (0.82)	30.9 (0.76)	31.8 (0.90)	0–100; higher score indicates better QoL
SF-36 MCS, mean (SE)	42.8 (1.09)	42.2 (1.26)	44.0 (1.11)	41.4 (1.14)	45.5 (1.29)	44.7 (1.23)	42.1 (1.29)	40.5 (1.31)	42.8 (1.32)	41.2 (1.23)	42.6 (1.17)	0–100; higher score indicates better QoL

*b.i.d.* twice daily; *HAQ-DI* Health Assessment Questionnaire-Disability Index, *FACIT* Functional Assessment of Chronic Illness Therapy, *ITT* intent-to-treat, *MCS* mental component summary score, *PCS* physical component summary score, *Patient Global* Patient Global Assessment of Disease Activity, *q.d.* once daily, *QoL* quality of life, *SE* standard error, *SF-36*, 36-Item Short Form Health Survey, *VAS* visual analog scale

### HAQ-DI

Changes from baseline in the HAQ-DI have been reported previously [11, 12]. To recapitulate, all treatment groups in both studies had a reduction in HAQ-DI scores over the course of the study (Table 2). At week 24 in the MTX add-on study, scores were better improved in the filgotinib-100 mg and 200-mg groups than in the placebo group (decrease of 0.66–0.90 points vs 0.37 points, respectively; *p* < 0.01). At week 12 in the monotherapy study, scores showed better improvement in the filgotinib 50-mg, 100-mg, and 200-mg groups than in the placebo group (decrease of 0.66 to 0.74 points vs 0.23 points, respectively; *p* < 0.001).

**Table 2:** Change from baseline in patient reported outcome scores at week 12 and week 24

Time-point	Methotrexate add-on study dosing group							Monotherapy study dosing group			
		Once daily (q.d.)			Twice daily (b.i.d.)			Once daily (q.d.)			
	Placebo	50	100	200	25	50	100	Placebo	50	100	200
Health Assessment Questionnaire-Disability Index (HAQ-DI), points											
Week 12	−0.38	−0.58	−0.65*	−0.75***	−0.59	−0.58	−0.84***	−0.23	−0.66***	−0.68***	−0.74***
Week 24	−0.37	−0.63	−0.78***	−0.82***	−0.62**	−0.66**	−0.90***	NA^a^	−0.69	−0.79	−0.85
Functional Assessment of Chronic Illness Therapy (FACIT)-Fatigue scale, points											
Week 12	5.6	7.6	9.5*	11.4***	6.9	8.4	11.3***	3.9	9.5***	10.2***	11.2***
Week 24	6.0	7.9	11.1***	11.6**	7.7	9.0	12.8***	NA^a^	10.0	11.3	13.7
Patient global assessment of disease activity (Patient Global), mm											
Week 12	−16.7	−25.2	−29.1*	−34.2***	−25.2*	−27.0*	−35.6***	−11.5	−27.5***	−30.0***	−28.2***
Week 24	−17.9	−29.4*	−34.4*	−34.9*	−27.3*	−28.1*	−39.1***	NA^a^	−29.1	−32.2	−35.1
Pain, mm											
Week 12	−16.9	−24.8	−27.4*	−31.4**	−24.2	− 28.3*	−37.9***	−13.3	−29.2***	−31.5***	−31.3***
Week 24	−17.0	−27.1*	−32.7***	−34.6***	−26.9*	−27.7*	−37.9***	NA^a^	−29.1	−35.1	−37.7
Short Form-36: Physical component score (PCS), points											
Week 12	3.2	6.7**	8.4***	8.9***	7.5**	7.1**	10.5***	3.0	7.1**	7.8***	8.6***
Week 24	2.8	7.3***	9.9***	9.7***	7.8***	7.9***	11.6***	NA^a^	6.9	10.0	9.7
Short Form-36: Mental component score (MCS), points											
Week 12	4.3	4.4	5.1	8.1	3.5	3.1	6.2	2.7	4.9*	6.9**	6.8**
Week 24	4.7	4.3	6.7	7.2	3.8	3.5	7.1	NA^a^	5.1	7.7	8.5

*b.i.d.* twice daily; *HAQ-DI* Health Assessment Questionnaire-Disability Index, *FACIT* Functional Assessment of Chronic Illness Therapy, *MCS* mental component summary score, *PCS* physical component summary score, *Patient Global* Patient Global Assessment of Disease Activity, *q.d.* once daily

^a^At week 12, patients receiving placebo in the add-on study and patients receiving placebo and filgotinib 50 mg q.d. in the monotherapy study who had not achieved a 20% improvement in swollen joint count and tender joint count were reassigned to receive filgotinib 100 mg q.d. (both studies) or 50 mg b.i.d. (methotrexate add-on study only). Patients who switched treatments at week 12 were handled as discontinuations and data were imputed from week 12 onwards using last observation carried forward

*P* values given for pair-wise comparison with placebo: *p < 0.05; **p < 0.01; ***p < 0.001

### Patient Global

**Fig. 1:**
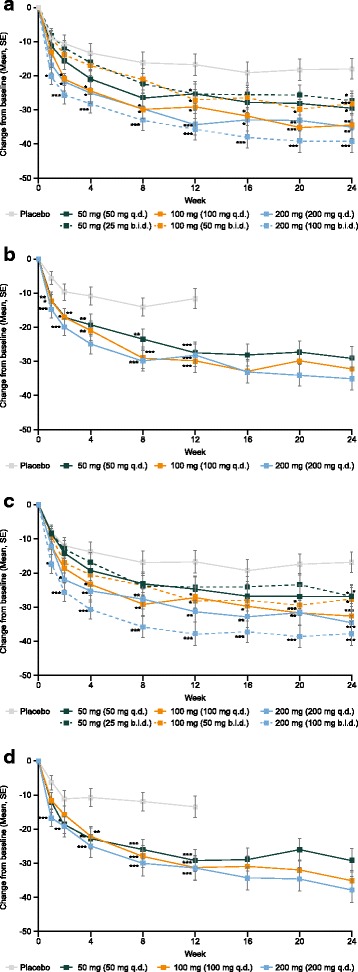
Patient Global scores, methotrexate (MTX) add-on (**a**) and monotherapy (**b**); Patient Pain scores, MTX add-on (**c**) and monotherapy (**d**). *P* values given for pair-wise comparison with placebo: *p < 0.05; **p < 0.01; ***p < 0.001. At week 12, patients receiving placebo in the MTX add-on study and patients receiving placebo and filgotinib 50 mg once daily (q.d.) in the monotherapy study, who had not achieved a 20% improvement in swollen joint count (SJC) and tender joint count (TJC) were reassigned to receive filgotinib 100 mg q.d. (both studies) or 50 mg twice daily (b.i.d.) (MTX add-on study). Patients who switched treatments at week 12 were handled as discontinuations and data were imputed from week 12 onwards using last observation carried forward. SE, standard error

In both studies, all treatment groups had a reduction in Patient Global scores over the 24-week period (Fig. 1a, b and Table 2). In the MTX add-on study, at week 24, scores had decreased by 17.9 mm in the placebo group and by 27.3–39.1 mm in the filgotinib groups. A statistically significantly greater improvement in Patient Global scores was noted as early as week 1 in the filgotinib 100-mg b.i.d. group versus placebo. By week 12, statistically significant improvements were seen in all but the 50-mg q.d. group versus placebo, and these improvements were sustained to week 24.

In the monotherapy study, scores at week 12 had decreased by 11.5 mm in the placebo group and by 27.5–30.0 mm in the filgotinib groups; at week 24, scores in the filgotinib groups were 29.1–35.1 mm lower than at baseline. Improvements in scores were statistically significant in all filgotinib groups versus placebo at week 1.

### Patient Pain

**Fig. 2:**
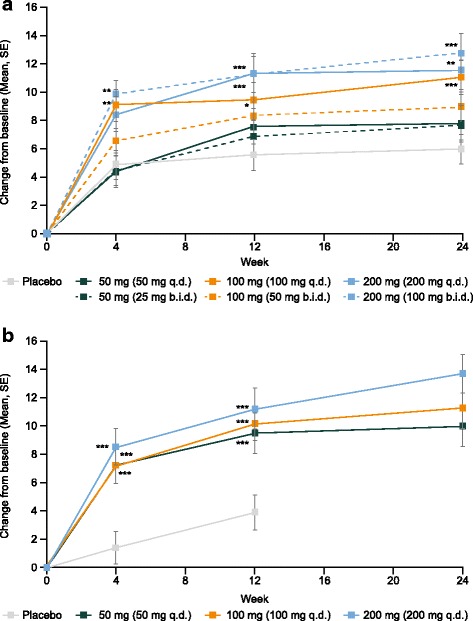
Functional Assessment of Chronic Illness Therapy (FACIT)-Fatigue, methotrexate (MTX) add-on (**a**) and monotherapy (**b**) studies. *P* values given for pair-wise comparison with placebo: *p < 0.05; **p < 0.01; ***p < 0.001. At week 12, patients receiving placebo in the MTX add-on study and patients receiving placebo and filgotinib 50 mg once daily (q.d.) in the monotherapy study who had not achieved a 20% improvement in swollen joint count (SJC) and tender joint count (TJC) were reassigned to receive filgotinib 100 mg q.d. (both studies) or 50 mg twice daily (b.i.d.) (MTX add-on study only). Patients who switched treatments at week 12 were handled as discontinuations and data were imputed from week 12 onwards using last observation carried forward. SE, standard error

In both studies, all placebo and filgotinib dosing groups had a reduction in Patient Pain scores over the course of the study (Fig. 2c, d and Table 2).

At week 24 in the MTX add-on study, scores had decreased by 17.0 mm in the placebo group and by 26.9–37.9 mm in the filgotinib groups. Statistically significantly greater improvements in pain versus placebo were noted as early as week 1 in the filgotinib 100-mg b.i.d. group, from week 2 in the filgotinib 200-mg q.d. group, and from week 4 in the 100-mg q.d. group (Fig. 2c).

In the monotherapy study, scores at week 12 had decreased by 13.3 mm in the placebo group and by 29.2–31.5 mm in the filgotinib groups; at week 24, scores in the filgotinib groups were 29.1–37.7 mm lower than at baseline. Statistically significantly greater improvements in Patient Pain scores were seen as early as week 1 in the filgotinib 200-mg q.d. group, by week 2 in the 50-mg q.d. group, and in all groups from week 4 to week 12 (Fig. 2d). Reductions in pain were maintained to week 24 in both studies.

### FACIT-Fatigue scale

In both studies, all treatment groups demonstrated numerical increases in FACIT-Fatigue scores over the course of the 24-week treatment period (Fig. 2 and Table 2), indicating reduced fatigue. At week 24 in the MTX add-on study, scores had increased by 6.0 points in the placebo group and by 7.7–12.8 points in the filgotinib groups. Statistically significantly greater improvements versus placebo were observed from the first measured time point—week 4—for filgotinib 100 mg q.d. and 100 mg b.i.d.. By week 12, improvements in the filgotinib 200-mg q.d. group were also statistically significantly greater than in the placebo group and remained so until week 24. There was no significant improvement in FACIT-Fatigue scores versus placebo at any time point in the filgotinib 50-mg b.i.d., 50-mg q.d., or 25-mg b.i.d. treatment groups (Fig. 2a).

In the monotherapy study, scores at week 12 were increased by 3.9 points in the placebo group and by 9.5–11.2 points in the filgotinib groups; at week 24, scores in the filgotinib groups were 10.0–13.7 points higher than at baseline. Statistically significantly greater improvements versus placebo were observed at week 4 in the monotherapy study in all filgotinib groups; these improvements versus placebo were maintained until week 12 and FACIT-Fatigue scores continued to increase to week 24 (Fig. 2b).

### SF-36

**Fig. 3:**
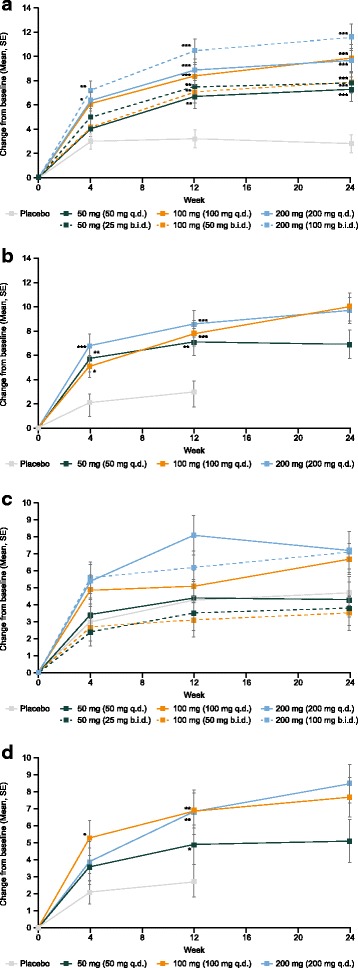
SF-36 physical component score (PCS), methotrexate (MTX) add-on (**a**) and monotherapy (**b**); SF-36 mental component score (MCS), MTX add-on (**c**), and monotherapy (**d**). *P* values given for pair-wise comparison with placebo: *p < 0.05; **p < 0.01; ***p < 0.001. At week 12, patients receiving placebo in the MTX add-on study and patients receiving placebo and filgotinib 50 mg once daily (q.d.) in the monotherapy study who had not achieved a 20% improvement in swollen joint count (SJC) and tender joint count (TJC) were reassigned to receive filgotinib 100 mg q.d. (both studies) or 50 mg twice daily (b.i.d.) (MTX add-on study only). Patients who switched treatments at week 12 were handled as discontinuations and data were imputed from week 12 onwards using last observation carried forward. SE, standard error; SF-36, 36-Item Short Form Health Survey

In both studies, improvements in both PCS and MCS scores were observed in all treatment groups (Fig. 3 and Table 2).

#### PCS

At week 24 in the MTX add-on study, PCS scores had increased by 2.8 points in the placebo group and by 7.3–11.6 points in the filgotinib groups. Statistically significant improvements versus placebo were observed at week 4 in the filgotinib 100-mg b.i.d. and 200-mg q.d. groups. By week 12, statistically significantly greater improvements were observed in all the filgotinib dosing groups compared with placebo, which continued to week 24 (Fig. 3a).

In the monotherapy study, PCS scores at week 12 were increased by 3.0 points in the placebo group and by 7.1–8.6 points in the filgotinib groups; at week 24, scores in the filgotinib groups were 6.9–10.0 points higher than at baseline. Statistically significantly greater improvements versus placebo were observed at week 4 in all filgotinib groups until week 12, and scores were maintained or improved further until week 24 (Fig. 3b).

#### MCS

In the add-on study, SF-36 MCS scores at week 24 had increased by 4.7 points in the placebo group and by 3.5–7.2 points in the filgotinib groups; however, these changes were not statistically significant in any filgotinib group (Fig. 3c).

Contrastingly, in the monotherapy study, there was a statistically significant difference in MCS in the filgotinib 100-mg q.d. group versus placebo at week 4 and in all filgotinib groups by week 12; SF-36 MCS scores remained stable or continued to improve until week 24 (Fig. 3d).

#### MCIDs

As detailed in Table 3, the placebo groups experienced MCIDs across PROs, ranging between 42% and 61% of patients at week 12; however, a numerically larger proportion of patients in the filgotinib groups achieved MCIDs at week 12 compared with placebo across all PROs for the vast majority of doses tested (range 45–86%; significance not determined). The three exceptions to this trend were patients in the MTX add-on study in the 50-mg q.d. group with respect to FACIT-Fatigue score and the 25-mg b.i.d. and 50-mg b.i.d. groups with respect to SF-36 MCS. The highest-dose groups in both studies were generally associated with the largest proportions of patients reporting MCIDs.

**Table 3:** Patients achieving MCIDs for each PRO at week 12

	Methotrexate add-on study							Monotherapy study (all q.d. doses)			
	Placebo (*N* = 86)	50 mg q.d. (*N* = 82)	100 mg q.d. (*N* = 85)	200 mg q.d. (*N* = 86)	25 mg b.i.d. (*N* = 86)	50 mg b.i.d. (*N* = 85)	100 mg b.i.d. (*N* = 84)	Placebo (*N* = 72)	50 mg (*N* = 72)	100 mg (*N* = 70)	200 mg (*N* = 69)
HAQ-DI, *n* (%)	49 (57)	60 (73)	64 (75)	74 (86)	61 (71)	57 (67)	70 (83)	37 (52)	51 (73)	55 (79)	55 (80)
Patient Global, *n* (%)	52 (61)	56 (68)	60 (71)	67 (78)	58 (67)	62 (73)	65 (77)	35 (49)	56 (78)	50 (71)	52 (75)
Patient Pain, *n* (%)	50 (58)	54 (66)	55 (66)	66 (77)	59 (69)	58 (68)	67 (80)	39 (55)	54 (77)	51 (73)	56 (81)
FACIT-Fatigue scale, *n* (%)	51 (59)	47 (57)	60 (71)	64 (74)	54 (63)	55 (65)	59 (71)	32 (45)	48 (69)	52 (74)	51 (74)
SF-36 PCS, *n* (%)	43 (50)	55 (67)	60 (71)	68 (79)	58 (67)	62 (73)	66 (80)	30 (42)	48 (69)	49 (70)	54 (78)
SF-36 MCS, *n* (%)	49 (57)	44 (54)	48 (57)	64 (74)	43 (50)	38 (45)	53 (64)	34 (48)	39 (56)	52 (74)	46 (67)

Minimal clinically important differences (MCIDs) were defined as follows: 0.22-point decrease from baseline in the Health Assessment Questionnaire–Disability Index (HAQ-DI); 10% (10 mm) decrease from baseline in the 'Patient' Global Assessment of Disease Activity and Patient Pain visual analog scale (VAS) scores; 4-point increase in the Functional Assessment of Chronic Illness Therapy (FACIT)-Fatigue score; 2.5-point increase from baseline in the 36-Item Short Form Health Survey (SF-36) physical component score (PCS) and mental component score (MCS)

*b.i.d.* twice daily, *PRO* patient-reported outcome, *q.d.* once daily

### Normative values

In an exploratory analysis of both studies, a numerically larger proportion of patients in each of the filgotinib groups achieved normalized PRO values at week 12 versus the placebo groups (Additional file 4: Table S3). The single exception was seen for SF-36 MCS, in which 38% of subjects in the placebo group of the MTX add-on study achieved scores ≥50 compared with 37% of patients in the 50-mg q.d. group.

### PRO responses by clinical response status

Patients who had not achieved a clinical response (ACR20) to treatment at week 12 and who were randomized from placebo or 50 mg filgotinib to 100 mg filgotinib daily experienced improvements in PRO scores from week 12 to week 24. At week 24, many of the scores reported by initial nonresponders were similar to those achieved by responders in the 100 mg filgotinib groups at week 12.

At week 12, patients who had demonstrated a clinical response to treatment in terms of ACR20 and ACR50 were numerically more likely to achieve normative PRO scores than nonresponders (Additional file 5: Figure S2; Additional file 6: Figure S3 and Additional file 7: Figure S4 for ACR20 responses (data not shown for ACR50 responses)). This pattern was observed consistently in both studies and across dose groups. Nonresponders in the placebo groups were numerically less likely to achieve normative PRO values compared with nonresponders in the filgotinib 100-mg and 200-mg groups for all PRO measures except SF-36 PCS.

### Dosing schedules

In both studies an overall dose effect was observed on all PROs (Table 2). In the MTX add-on study, after 24 weeks of treatment, there appeared to be a numerically better response with filgotinib 100 mg q.d. compared with 50 mg b.i.d. across all PROs (Figs. 1, 2 and 3). In contrast, numerically greater improvements were observed with filgotinib 100 mg b.i.d. compared with 200 mg q.d. across all PROs, with the exception of SF-36 MCS scores. Only the SF-36 MCS scores were significantly different between the 100-mg q.d. and 50-mg b.i.d. groups (least squares mean difference, 2.9; 95% confidence interval, 0.4 to 5.4; *p* = 0.0249 (uncorrected *p* value)). There were no apparent differences between dosing regimens with the 50-mg daily dose.

## Discussion

Two placebo-controlled studies assessed the efficacy and safety of filgotinib in combination with MTX or as monotherapy. Over 24 weeks of treatment, filgotinib provided statistically significant improvements versus placebo across measured PROs, covering a range of aspects of patient HRQoL, including pain, functional status, physical wellbeing, and fatigue. There was also a trend towards improvements in mental health, which was statistically significant compared to placebo in the monotherapy study. A rapid onset of effect was observed for improvements in HRQoL with the active treatment. Onset of effect was generally faster in the higher dose groups: a significantly greater improvement in Patient Global and Patient Pain scores was seen at week 2 in the filgotinib 200-mg groups versus placebo in both studies, and in the FACIT-Fatigue and SF-36 PCS at the first measured time point (week 4). Furthermore, patients in the 200-mg filgotinib groups in both studies were numerically more likely to achieve MCIDs across PROs versus patients on placebo. Similarly, the active treatment was associated with a numerically larger proportion of patients achieving normative PRO scores than placebo patients in all but one outcome. PRO scores appeared to mirror measures of clinical efficacy as ACR20 and ACR50 responders were numerically more likely to achieve normative PRO scores than nonresponders.

This analysis was not powered to compare filgotinib dose groups; however, an exploratory analysis was performed to better inform regimen choices in future studies. In the MTX add-on study, in which both q.d. and b.i.d. dosing regimens were tested, there were numerically better responses with a daily dose of filgotinib 100 mg when given as a 100-mg q.d. dose versus a 50-mg b.i.d. dose across all PROs, although this only reached statistical significance in one outcome (SF-36 MCS). Conversely, with a daily dose of filgotinib 200 mg, treatment response appeared to be higher with a 100-mg b.i.d. dose versus a 200-mg q.d. dose across all PROs, with the exception of the SF-36 MCS.

The strong placebo effect cannot be overlooked. For example, at week 12, 61% of patients in the MTX add-on study and 49% of patients in the monotherapy study receiving placebo reported a MCID in terms of Patient Global scores. As well as being striking in itself, the placebo effect may have influenced differences in results between the two studies. For example, whereas all filgotinib groups in the monotherapy study had statistically significant improvements in SF-36 MCS scores versus placebo, in the MTX add-on study, there were no such significant improvements in any active treatment group. These divergent findings may be explained by the administration of MTX in the add-on study, thus the “placebo” group continued to receive an active treatment, which may underlie the rapid increase in SF-36 scores in this group (versus more modest gains in the placebo group of the monotherapy study). This also highlights the complex nature of the psychological burden of RA, suggesting that patient emotional wellbeing is affected by factors beyond the treatment itself. It is notable that the placebo response in this study is in line with that observed during studies of other RA therapies [24–29].

The limitations of these studies include the inherent subjectivity of PRO measures, although PROs do give valuable information on the treatment effect. Patients may have experienced inflated improvements in their health status as a result of the more attentive care and close monitoring received in clinical trials or as a result of high expectations of novel treatments, as suggested by the placebo effect noted above. The switching of all patients in the placebo group in the monotherapy study, and nonresponders in the placebo and filgotinib 50-mg groups in the MTX add-on study, means that placebo control was maintained for the first 12 weeks only (owing to the ethical implications of leaving patients without effective treatment). Furthermore, as the overall studies were of 6-month duration, the longer-term impact of filgotinib treatment remains to be determined.

## Conclusions

In both studies, rapid and sustained improvements in PROs during 24 weeks of treatment were observed when filgotinib was given in combination with MTX or as monotherapy, and as a q.d. or b.i.d. dosing regimen, to patients with moderate-to-severe active RA. The clinical benefits of filgotinib previously reported [11, 12] gain added value when combined with patient insights such as these, which confirm the potential ability of filgotinib to improve patients’ HRQoL as well as the signs and symptoms of disease.

## Additional files

Additional file 1: **Figure S1.** Patient disposition during the methotrexate (MTX) add-on study (A) and the monotherapy study (B). (ZIP 1513 kb)

Additional file 2: Document 1. List of ethical bodies that approved the DARWIN 1 study for each of the 106 study centers. (PDF 129 kb)

Additional file 3: Document 2. List of ethical bodies that approved the DARWIN 2 study for each of the 59 study centers. (PDF 68 kb)

Additional file 4: **Table S1.** Patient-reported outcomes: summary of instruments used and minimally important clinical differences used in this study. **Table S2.** Baseline patient characteristics. **Table S3.** Proportion of patients who achieved normative scores at week 12 in the methotrexate add-on and monotherapy studies. (DOCX 34 kb)

Additional file 5: **Figure S2.** Proportion of subjects achieving normative HAQ-DI score (≤0.5) at week 12 according to ACR20 responder status in the methotrexate (MTX) add-on study (A) and monotherapy study (B). (EPS 1981 kb)

Additional file 6: **Figure S3.** Proportion of subjects achieving normative FACIT-Fatigue score (≥40) at week 12 according to ACR20 responder status in the MTX add-on study (A) and monotherapy study (B). (EPS 1984 kb)

Additional file 7: **Figure S4.** Proportion of subjects achieving normative SF-36 PCS and MCS scores (≥50) at week 12 according to ACR20 responder status. A PCS, MTX add-on, B PCS, monotherapy, C MCS, MTX add-on, D MCS, monotherapy. (EPS 2249 kb)

## Abbreviations

ACR: American College of Rheumatology
bDMARDs: Biologic DMARDs
b.i.d: Twice daily
FACIT: Functional Assessment of Chronic Illness Therapy
HAQ-DI: Health Assessment Questionnaire-Disability Index
HRQoL: Health-related quality of life
ITT: Intent-to-treat
JAK: Janus kinase
MCIDs: Minimal clinically important differences
MCS: Mental component summary
MTX: Methotrexate
PCS: Physical component summary
PRO: Patient-reported outcome
q.d.: Once daily
QoL: Quality of life
RA: Rheumatoid arthritis
SE: Standard error
SF-36: 36-Item Short Form Health Survey
SJC: Swollen joint count
TJC: Tender joint count
tsDMARDs: Targeted synthetic disease-modifying antirheumatic drugs
VAS: Visual analog scale
