# Supplementary material for: Effect of filgotinib, a selective JAK 1 inhibitor, with and without methotrexate in patients with rheumatoid arthritis: patient-reported outcomes
Source: Arthritis Res Ther. 2018 Mar 23;20:57. doi: 10.1186/s13075-018-1541-z (PMC5865354; doi:10.1186/s13075-018-1541-z)
Supplement: Supplementary file 2 — Document 1. List of ethical bodies that approved the DARWIN 1 study for each of the 106 study centers. (PDF 129 kb) [file 13075_2018_1541_MOESM2_ESM.pdf]

Country	Central EC Name and Address	Local EC Name and Address (repeat as necessary)
Argentina	NA	Site 0102 (Dr. Cappuccio)<br>"Comité de ética en Investigación Clínica (CEIC)<br>Address: Larrea 1381 3° A. (C1117ABK) C.A.B.A. Argentina"<br><br>Site 0103 (Dr. Lázaro)<br>"Comité de ética San Isidro<br>Address:Av. Del Libertador 16958, San Isidro, Pcia. De Buenos Aires. Argentina"<br><br>Site 0105 (Dr. Spindler)<br>"Comité Independiente de Ética del Noroeste Argentino<br>Address: Las Piedras 496, 4° Floor, San Miguel de Tucumán, Argentina"<br><br>Site 0106 (Dr. Strusberg)<br>"Comité de ética Instituto Reumatológico Strusberg<br>Address: Aav. E. Olmos 247 1° Floor, Córdoba, Argentina"<br><br>Site 0107 (Dr. Tate)<br>"Comité de ética en Investigación Clínica (CEIC)<br>Address: Larrea 1381 3° A. (C1117ABK) C.A.B.A. Argentina"<br><br>Site 0108 (Dr. Velasco)<br>"Comité de ética de CER Investigaciones Clínicas<br>Address: Vicente López 1441, Quilmes, Pcia. De Buenos Aires. Argentina "
Australia	Metro South Human Research Ethics Committee<br>Centres for Health Research<br>Princess Alexandra Hospital<br>Woolloongabba QLD 4102	Southern Adelaide Clinical<br>Human Research Ethics Committee The Flats G5 – Rooms 3 and 4<br>Flinders Drive<br>Flinders Medical Centre, Bedford Park SA 5042
Austria	City of Vienna Ethics Committee, Thomas-Klestil-Platz 8, TownTown 1st Floor, CB 12.103 , 1030, Venice	NA
Belgium	**UZ Leuven**<br>**EC:** Commissie Medische Ethiek<br>Universitaire Ziekenhuizen KULeuven<br>Address: Campus Gasthuisberg<br>Herestraat 49<br>3000 Leuven	**1)CHU LIÈGE**<br>**EC:** Comité d'Ethique de CHU Liège<br>Address :Centre Hospitalier Universitaire de Liège, Domaine universitaire du Sart Tilman, Bâtiment B35, 4000 Liège, Belgium<br>**2)AZ GROENINGEN**<br>**EC:** Ethisch Comité voor klinische studies, AZ Groeninge<br>Address : Loofstraat 43, 8500 Kortrijk, Belgium<br>**3)CLINIQUES UNIVERSITAIRES SAINT-LUC**<br>**EC:** Commission d'Ethique Biomédicale Hospitalo-Facultaire de l'UCL<br>Address: avenue Hippocrate 55.14, 1200 Bruxelles Belgium<br>**4)CHU BRUGMANN**<br>**EC:** COMITE D'ETHIQUE du CHU BRUGMANN<br>Address: Place A. Van Gehuchten 4 ; 1020 Bruxelles Belgium<br>**5)REUMA INSTITUUT HASSELT**<br>**EC:** Commissie Medische Ethiek, Universitaire Ziekenhuizen K.U. Leuven, Belgium<br>(Refer to the address of the CEC UZ Leuven)
Bulgaria	Ethics Committee for Multicenter Trials, 5, Sveta Nedelya, Sq, 1000 Sofia, Bulgaria	NA

Chile	NA	Site 0601 - Aliste<br>Comite de Etica Cientifico Servicio de Salud Metropolitano Oriente<br>Av. Salvador 364, Santiago, RM, Chile, 7500922<br><br>Site 0602 - Baumert<br>Comite de Etica Cientifico del Servicio de Salud Araucania Sur<br>Andres Bello # 636, Temuco, IX Region, Chile, 4791301<br><br>Site 0603 - Goecke<br>Comite de Etica Cientifico Servicio de Salud Metropolitano Oriente<br>Av. Salvador 364, Santiago, RM, Chile, 7500922<br><br>Site 0604 - Holuigue<br>Comite de Etica Cientifico Servicio de Salud Metropolitano Oriente<br>Av. Salvador 364, Santiago, RM, Chile, 7500922<br><br>Site 0605 - Ponce<br>Comite de Etica Cientifico del Servicio de Salud Araucania Sur<br>Andres Bello # 636, Temuco, IX Region, Chile, 4791301<br><br>Site 0606 - Hernandez<br>Comite de Etica Cientifico del Servicio de Salud Conception<br>San Martin 1436, Ceoncepcion, VIII Region, Chile, 4070038
Colombia	NA	0701 - Comité de ética en Investigación de la División Ciencias de la Salud de la Universidad del Norte<br>Km 5 Via Puerto Colombia Barranquilla - Colombia<br><br>0703 - 704 - 706 - 708 Comité de Ética de Investigación Riesgo de Fractura S.A<br>Carrera 13 No. 97-25 Bogotá - Colombia<br><br>0705 - Comité de Investigaciones y Ética en Investigaciones del Hospital Pablo Tobón Uribe<br>Calle 78 B No. 69-240 Medellin - Colombia<br><br>0707 - Comité de Ética en Investigaciones del Oriente<br>Calle 53 No. 34-20 Bucaramanga - Colombia<br><br>0709 - Comité de ética en investigación de la Fundación del Caribe para la Investigación BiomedicaCarrera 50 No. 80-216 Barranquilla - Colombia<br><br>0710 - Comité de ética en investigación del centro médico Imbanaco<br>Cra. .8A No. 5A-100 Cali - Colombia
Czech Republic	Eticka komise FN Brno, Jihlavska 20, 625 00 Brno, Czech Republic	Etiská komise Revmatologického ústavu, Na Slupi 4, 12800 Praha 2, Czech Republic
France	CPP Sud Mediterranee III, UFR medicine, 186 Chemin du Carreau de Lanes, CS-83021, Nimes Cedex 2, France, 30908	NA

Germany	Landesamt für Gesundheit und Soziales,<br>Geschäftsstelle der Ethik-Kommission<br>des Landes Berlin<br>Fehrbelliner Platz 1<br>D-10707 Berlin	Ethik-Kommission der Ärztekammer Hamburg<br>Weidestr. 122b<br>D-22083 Hamburg<br><br>Ethik-Kommission des Fachbereichs Medizin<br>der Johann Wolfgang Goethe-Universität<br>Haus 1, 2. OG, Zi. 222/223<br>Theodor-Stern-Kai 7<br>D-60596 Frankfurt am Main<br><br>Ethik-Kommission der Ärztekammer<br>Westfalen-Lippe und der Medizinischen Fakultät der<br>Westfälischen Wilhelms-Universität Münster<br>Gartenstr. 210 - 214<br>D-48147 Münster<br><br>Ethik-Kommission der Medizinischen Fakultät<br>und am Universitätsklinikum Tübingen<br>Gartenstr. 47<br>D-72074 Tübingen
Guatemala	NA	Comité Independiente de Ética Latin Ethics<br><br>Comité de Ética Independiente ZUGUEME<br><br>Comité de Ética en Investigación del Hospital Centro Médico
Hungary	Hun: Egészségügyi Tudományos Tanács Klinikai Farmakológiai Etikai Bizottsága<br>Eng: Medical Research Council Ethics Committee for Clinical Pharmacology<br>Address: 1051 Budapest, Arany J. u. 6-8, Hungary	NA
Israel	NA	Dr. Lorber - Local EC, Rambam MC<br>Dr. Lidar - Local EC, Sheba MC<br>Dr. Zisman/Feld - Local EC, Carmel MC
Latvia	Independent Ethics Committee for Investigation of Drugs and Pharmaceutical Products, A. Briana street 2, Riga, LV-1001, Latvia	NA

Mexico	NA	Site 1501 - De La Garza Ramos<br>Comite de Etica en Investigacion<br>Modesto Arreola 917 Ote. Monterrey, Nuevo Leon, Mexico, 64000<br><br>Site 1502 - Enriquez Sosa<br>Comite Vioetico para la Investigacion Clinica (CBIC)<br>Puebla 422 int 4, Col Roma Sur, Mexico, DF, 06700<br><br>Site 1503 - Flores Alvarado<br>Comite de Etica en Investigacion de la facultatd de Medicina y Hospital Universitario de la Universidad Autonoma de Nuevo Leon<br>Av. Francisco I Medero y Dr Eduardo Aguirre Pequeno, Col. Mitras Centre, Monterray, Nuevo leon, Mecixo, 64460<br><br>Site 1504 - Garcia de la Torre<br>Comite Independiente de Etica e Investigacion del Centro de Estudios de Investigacion Basica y Clinica SC, Justo Sierra 2821-4, Col Vallarta Norte, Guadalajara, Jalisco, Mexico, 44690<br><br>Site 1505 -Garcia Garcia<br>Comite de Etica en Investigacion del Hospital General de Mexico "Dr Eduardo Liceaga"<br>Dr Baalmis No 148, Col Doctores, Distrito Federal, Mexico, 06726<br><br>Site 1507 - Rizo Rodriguez<br>Comite Bioetico para la Investigacion Clinica<br>Peubla No 422 - 4, Col Roma, DF, Mexico, 06700<br><br>Site 1509 - Vicente Gonzalez<br>omite Vioetico para la Investigacion Clinica (CBIC)
Moldova	National Ethics Committee Clinical Research of Drugs and new Methods of Treatment, Testemitanu Str 27, Chisinau, Moldova, MD2020	NA
New Zealand	Northern B Health and Disability Ethics Committee 20 Aitken Street<br>PO Box 5013<br>Wellington	NA
Poland	Komisja Bioetyczna przy Okręgowej Radzie Lekarskiej<br>Wielkopolskiej Izby Lekarskiej<br>*[Bioethics Committee at the Regional Medical Council of the Greater Poland Medical Chamber]*<br>Ul. Nowowiejskiego 51; 61-734 Poznań	NA

Russia	Ethics Council under the Ministry of Health<br>of the Russian Federation<br>3, Rakhmanovsky per.<br>127994, Moscow	Site(s) 2101/2103 - Inter-Collegiate ETHICS COMMITTEE. 37 Gagarinsky per., bldg. 2, Moscow 119002<br><br>Site 2104 - Ethics Committee Municipal Clinical Hospital No. 5<br>of Nizhny Novgorod District, Nizhny Novgorod, a state-funded healthcare facility of Nizhny Novgorod Oblast (GBUZ NO) 34 ul. Nesterova, Nizhny Novgorod 603005;<br><br>Site 2105 - ETHICS COMMITTEE, at Regional Clinical Hospital, a state-funded healthcare facility of Vladimir Region, 41 Sudogodskoye Shosse, Vladimir 600023,<br><br>Site 2107 - COMMITTEE FOR HEALTHCARE<br>GOVERNMENT OF ST. PETERSBURG<br>ST. PETERSBURG STATE BUDGETARY HEALTHCARE<br>INSTITUTION "MUNICIPAL CLINICAL HOSPITAL No. 26 2 Kostyushko St., St. Petersburg 196247<br><br>Site 2108 - Ethics Committee at FGBU V.A. Nasonova NIIR RAMN 34A Kashirskoye Shosse, Moscow 115522<br><br>Site 2109 - REGIONAL CLINICAL CARDIOLOGY DISPENSARY (OKKD), A PUBLICLY-FUNDED
Spain	PI Dr. Antonio Gómez Centeno - CEIC de la Corporació Sanitària Parc Taulí<br>Fundació Parc Taulí<br>Edificio Santa Fe<br>Ala izquierda, 2ª planta<br>Parc Taulí, núm. 1<br>08208 Sabadell (Barcelona)	CEIC de Galicia (SERGAS) - PI Dr. Francisco Javier Blanco García<br>COMITÉ ÉTICO DE INVESTIGACIÓN CLÍNICA DE GALICIA<br>División de Farmacia y Productos Sanitarios/Servicio Gallego de Salud<br>Edificio Administrativo San Lázaro<br>15781 Santiago de Compostela- A Coruña<br><br>CEIC Hospital General Universitario de Elche - PI Dr. José Antonio González<br>Hospital General Universitario de Elche<br>Comité Ético de Investigación Clínica<br>(3ª Planta edificio Anexo II)<br>C/. Camí de L'Almazara, 11<br>03203 – Elche (Alicante)<br><br>Secretaría del Comité Ético de Investigación Clínica (C.E.I.C) - PI Dra. Jacqueline Usón Jaeger<br>Hospital Universitario de Móstoles<br>C/ Río Júcar, s/n - 2ª planta<br>28935 Móstoles (Madrid)<br><br>Comité Coordinador de Ética de la Investigación Biomédica de Andalucía - PI Dr. Alejandro Escudero<br>Consejería de Igualdad, Salud y Políticas Sociales Avda Innovación, s/n Edif. Arena 1<br>Sevilla CP: 41020<br><br>Comité Coordinador de Ética de la Investigación Biomédica de Andalucía - PI Dra. Virginia Moreira Navarrete<br>Consejería de Igualdad, Salud y Políticas Sociales Avda Innovación, s/n Edif. Arena 1<br>Sevilla CP: 41020<br><br>Comité Ético de Investigación Clínica de Asturias - PI Dra. Mercedes Alperi López<br>5ª Planta Centro de Rehabilitación<br>Celestino Villamil, s/n<br>33006 Oviedo

Ukraine	NA	Site 2201 - Gasanov<br>Medical Ethics Comittee of GI "L.T. Malaya Therapy National Institute of the National Academy of Medical Sciences of Ukraine"<br>2-A Postysheva Avenue, Kharkiv, Ukraine, 61039<br><br>Site 2202 - Gnylorybov<br>Bioethics Comittee of State Institution "Institute of Urgent and Recovery Surgery n.a V.K Gusak NAMS of Ukraine"<br>47 Leninsky Avenue, Donetsk, Ukraine, 83045<br><br>Site 2204 - Lymar<br>Local Ethics Comittee of Municipal Non-Profit Institution "Consultative and Diagnostic Centre" of Desynansky District of Kyviv<br>81/1 Zakrevskogo Str, Kyiv, Ukraine, 02232<br><br>Site 2205 - Shpyleva<br>Ethics Comittee of Municipal Healthcare Instiution "City Hospital #5 of Donetsk City"<br>88 Krasnoarmeyskaya Stre, Donetsk, Ukraine, 83000<br><br>Site 2207 - Trubina<br>Ethics Comittee of Municipal Non-Profit Institution "Consultative and Diagnostic Centre" of Perchersky District of Kyiv<br>13 Podvysotskogo str, Kyiv, Ukraine 01103<br><br>Site 2208 - Tseluyko<br>Ethics Committee of Municipal Healthcare Institution "Kharkiv City Clinical Hospital #8"<br>266 G Saltivske Shose, Kharkiv, Ukraine, 61178<br><br>Site 2209 - Voyeykova<br>Ethics Comittee of Communal Institution of Healthcare "Kharkiv City Clinical Hospital No 13"<br>137 Gagarina Avnue, Kharkiv, Ukraine, 61124<br><br>Site 2210 - Yagensky<br>Ethics Comittee of Municipal Institution "Lutsk City Clinical Hospital"<br>13 Vidrodzhennya pr, Lutsk, Ukraine, 43024
USA	Copernicus IRB (CGIRB)	• Western IRB (WIRB) for Mease Site # 2330<br>1019 39th Avenue South East, Puyallup, WA, USA, 98502<br>• UCSD IRB for Lee site # 2313<br>9500 Gilman Drive, La Jolla, CA, USA, 92093-0052<br>• Mayo Clinic IRB Michet site # 2319<br>200 1st Street SW, Rochester, MN, USA, 55905
