# Supplementary material for: Effect of filgotinib, a selective JAK 1 inhibitor, with and without methotrexate in patients with rheumatoid arthritis: patient-reported outcomes
Source: Arthritis Res Ther. 2018 Mar 23;20:57. doi: 10.1186/s13075-018-1541-z (PMC5865354; doi:10.1186/s13075-018-1541-z)
Supplement: Supplementary file 3 — Document 2. List of ethical bodies that approved the DARWIN 2 study for each of the 59 study centers. (PDF 68 kb) [file 13075_2018_1541_MOESM3_ESM.pdf]

Country	Central EC Name and Address	Local EC Name and Address (repeat as necessary)
Argentina	NA	(Site 0101) Dr. Brea<br>"Comité de Bioética C.I.M.E.L.<br>Address: Tucumán 1314, Lanús Este, Pcia. De Buenos Aires, Argentina"<br><br>(Site 0104) Dr. Machado<br>"Comité de Ética CAICI-CIAP<br>Address: Rodríguez 1198 (2000) Rosario, Santa Fe, Argentina"<br><br>(Site 0105) Dr. Spindler<br>"Comité Independiente de Ética del Noroeste Argentino<br>Address: Las Piedras 496, 4° Floor, San Miguel de Tucumán, Argentina"
Australia	Metro South Human Research Ethics Committee Centres for Health Research<br>Princess Alexandra Hospital<br>Woolloongabba QLD 4102	
Austria	City of Vienna Ethics Committee, Thomas-Klestil-Platz 8, TownTown 1st Floor, CB 12.103 , 1030, Venice	NA
Bulgaria	Ethics Committee for Multicenter Trials, 5, Sveta Nedelya, Sq, 1000 Sofia, Bulgaria	NA
Chile	NA	Site 0604 - Holuigue<br>Comite de Etica Cientifico Servicio de Salud Metropolitano Oriente<br>Av. Salvador 364, Santiago, RM, Chile, 7500922<br><br>Site 0605 - Ponce<br>Comite de Etica Cientifico del Servicio de Salud Araucania Sur<br>Andres Bello # 636, Temuco, IX Region, Chile, 4791301<br><br>Site 0606 - Hernandez<br>Comite de Etica Cientifico del Servicio de Salud Conception<br>San Martin 1436, Ceoncepcion, VIII Region, Chile, 4070038
Colombia	NA	0703 - 704 - 706 - 708 Comité de Ética de Investigación Riesgo de Fractura S.A<br>Carrera 13 No. 97-25 Bogotá - Colombia<br>0707 - Comité de Ética en Investigaciones del Oriente<br>Calle 53 No. 34-20 Bucaramanga - Colombia<br>0709 - Comité de ética en investigación de la Fundación del Caribe para la Investigación Biomedica<br>Carrera 50 No. 80-216 Barranquilla - Colombia
Germany	Landesamt für Gesundheit und Soziales,<br>Geschäftsstelle der Ethik-Kommission<br>des Landes Berlin<br>Fehrbelliner Platz 1<br>D-10707 Berlin	Ethik-Kommission der Ärztekammer Hamburg<br>Weidestr. 122b<br>D-22083 Hamburg
Guatemala	NA	Comité Independiente de Ética Latin Ethics
Hungary	Hun: Egészségügyi Tudományos Tanács Klinikai Farmakológiai Etikai Bizottsága<br>Eng: Medical Research Council Ethics Committee for Clinical Pharmacology<br>Address: 1051 Budapest, Arany J. u. 6-8, Hungary	NA

Latvia	Independent Ethics Committee for Investigation of Drugs and Pharmaceutical Products, A. Briana street 2, Riga, LV-1001, Latvia	NA
Mexico	NA	Site 1502 - Enriquez Soza<br>Comite Vioetico para la Investigacion Clinica (CBIC)<br>Puebla 422 int 4, Col Roma Sur, Mexico, DF, 06700<br><br>Site 1503- Flores Alvarado<br>Comite de Etica en Investigacion de la facultatd de Medicina y Hospital Universitario de la Universidad Autonoma de Nuevo Leon<br>Av. Francisco I Medero y Dr Eduardo Aguirre Pequeno, Col. Mitras Centre, Monterray, Nuevo leon, Mecixo, 64460<br><br>Site 1506 - Paz Viscarra<br>Comite Bioetico para la Investigacion Clinica (CBIC)<br>Puebla 422 int 4, Col Roma Sur, Mexico, DF, 06700<br><br>Site 1509 - Vicente Gonzalez<br>Comite Bioetico para la Investigacion Clinica (CBIC)<br>Puebla 422 int 4, Col Roma Sur, Mexico, DF, 06700<br><br>Site 1510 - Araujo Arias<br>Tuxpan 29, Interior 704, Col, Roma Sur Distrito Federal, Mexico, 06760<br><br>Site 1511 - Garcia Olivera<br>Comite de Etica en Investigacion de Oaxaca Site Management Organiszation SC<br>Humboldt 302, Col Centro, Oaxaxo, Mexico, 68000
Moldova	National Ethics Committee Clinical Research of Drugs and new Methods of Treatment, Testemitanu Str 27, Chisinau, Moldova, MD2020	
New Zealand	Northern B Health and Disability Ethics Committee 20 Aitken Street<br>PO Box 5013<br>Wellington	NA
Poland	"Komisja Bioetyczna przy Okręgowej Radzie Lekarskiej<br>Wielkopolskiej Izby Lekarskiej<br>[Bioethics Committee at the Regional Medical Council of the Greater Poland Medical Chamber]<br>Ul. Nowowiejskiego 51; 61-734 Poznań	NA
Romania	National Bioethics Committee for Medicines and Medical Devices<br>Sos. Stefan cel Mare 19-21, district 2<br>020125, Bucharest, Romania (ENGLISH LANGUAGE)<br><br>Comisia Nationala de Bioetica a Medicamentului si a Dispozitivelor Medicale<br>Sos. Stefan cel Mare 19-21, sector 2<br>020125, Bucuresti, Romania (ROMANIAN LANGUAGE)	NA

Russia	Ethics Council under the Ministry of Health<br>of the Russian Federation<br>3, Rakhmanovsky per.<br>127994, Moscow	Site 2102 - LOCAL ETHICS COMMITTEE AT ORENBURG STATE MEDICAL UNIVERSITY(OrGMU), A STATE-FUNDED INSTITUTION of HIGHER PROFESSIONAL EDUCATION (GBOU VPO) OF THE MINISTRY OF HEALTH OF THE RUSSIAN FEDERATION<br>6 ul. Sovietskaya, Orenburg 460000<br><br>Site 2105 - ETHICS COMMITTEE, at Regional Clinical Hospital, a state-funded healthcare facility of Vladimir Region, 41 Sudogodskoye Shosse, Vladimir 600023,<br><br>Site 2106 - GUZ (State Healthcare Institution) “OKB” (“Regional Clinical Hospital) LOCAL ETHICS COMMITTEE 1 Smirnovskoye Ushchelye, Saratov, 410053 "
Spain	CEIC de la Corporació Sanitària Parc Taulí - PI Dr. Antonio Gómez Centeno<br>Fundació Parc Taulí<br>Edificio Santa Fe<br>Ala izquierda, 2ª planta<br>Parc Taulí, núm. 1<br>08208 Sabadell (Barcelona)	CEIC de Galicia (SERGAS) -PI Dr. Manuel Enrique Pombo Suárez.<br>COMITÉ ÉTICO DE INVESTIGACIÓN CLÍNICA DE GALICIA<br>División de Farmacia y Productos Sanitarios/Servicio Gallego de Salud<br>Edificio Administrativo San Lázaro<br>15781 Santiago de Compostela- A Coruña<br><br>CEIC Hospital General Universitario de Elche - PI Dr. José Antonio González<br>Hospital General Universitario de Elche<br>Comité Ético de Investigación Clínica<br>(3ª Planta edificio Anexo II)<br>C/. Camí de L’Almazara, 11<br>03203 – Elche (Alicante)<br><br>Comité Ético de Investigación Clínica de Asturias - PI Dra. Mercedes Alperi López<br>5ª Planta Centro de Rehabilitación<br>Celestino Villamil, s/n<br>33006 Oviedo

Ukraine	NA	Site 2202 - Gnylorybov<br>Bioethics Comittee of State Institution "Institute of Urgent and Recovery Surgery n.a V.K Gusak NAMS of Ukraine"<br>47 Leninsky Avenue, Donetsk, Ukraine, 83045<br><br>Site 2203 - Golovchenko<br>Local Ethics Committee of Municipal Institution "Kherson City Clinical Hospital n.a.A and O.Tropins"<br>2 Komarova str, Kherson, Ukraine, 73000<br><br>Site 2204 - Lymar<br>Local Ethics Comittee of Municipal Non-Profit Institution "Consultative and Diagnostic Centre" of Desynansky District of Kyviv<br>81/1 Zakrevskogo Str, Kyiv, Ukraine, 02232<br><br>Site 2206 - Stanislavchuk<br>Ethics Comittee of Vinnitsya Regional Clinical Hospital Named after M.I.Pirogov<br>46 Pirogova str, asd, Vinnitsya, Ukraine, 21018<br><br>Site 2208 - Tseluyko<br>Ethics Committee of Municipal Healthcare Institution "Kharkiv City Clinical Hospital #8"<br>266 G Saltivske Shose, Kharkiv, Ukraine, 61178
USA	Copernicus IRB (CGIRB)	• UCSD IRB for Lee site # 2313<br>9500 Gilman Drive, La Jolla, CA, USA, 92093-0052
