# Supplementary material for: Effect of filgotinib, a selective JAK 1 inhibitor, with and without methotrexate in patients with rheumatoid arthritis: patient-reported outcomes
Source: Arthritis Res Ther. 2018 Mar 23;20:57. doi: 10.1186/s13075-018-1541-z (PMC5865354; doi:10.1186/s13075-018-1541-z)
Supplement: Supplementary file 4 — Table S1. Patient-reported outcomes: summary of instruments used and minimally important clinical differences used in this study. Table S2. Baseline patient characteristics. Table S3. Proportion of patients who achieved normative scores at week 12 in the methotrexate add-on and monotherapy studies. (DOCX 34 kb) [file 13075_2018_1541_MOESM4_ESM.docx]

## Additional file 4

**Table S1.** Patient-reported outcomes: summary of instruments used and minimally important clinical differences used in this study

| **Instrument** | **Description** | **Quantification** | **Minimally important clinical difference** | **Normative value** |
| --- | --- | --- | --- | --- |
| Health Assessment Questionnaire-Disability Index (HAQ-DI)[1] | Specifically designed to measure functional status in patients with arthritis by assessing the degree of difficulty a person has in accomplishing tasks in eight domains:   1. Dressing 2. Getting up (arising) 3. Eating 4. Walking 5. Hygiene 6. Reaching 7. Gripping 8. Errands/chores | Responses scored on a 4-point Likert scale from 0, indicating no difficulty, to 3, indicating inability to perform a task in that area  Final HAQ-DI score calculated by taking the mean across the eight domains, producing a value between 0 and 3, where a higher number indicates greater disability | 0.22‑point decrease from baseline | ≤0.5 |
| Patient Pain (assessed as part of the HAQ-DI) | Assesses average pain over the previous week | 0- to 100-mm visual analog scale, with 0 indicating ‘no pain’ and 100 indicating ‘severe pain’ | 10% (10-mm) decrease from baseline | - |
| Patient’s Global Assessment of Disease Activity (Patient Global) | Evaluates patient well-being in response to a specific question about their rheumatoid arthritis:  “Considering all the ways arthritis affects you, how well are you doing today?” | 100-mm visual analog scale, where 0 indicates ‘very well’ and 100 indicates ‘very poor’; higher scores indicate greater impairment | 10% (10-mm) decrease from baseline | - |
| Functional Assessment of Chronic Illness Therapy (FACIT)-Fatigue scale [2, 3] | Measures level of fatigue over a 1-week period during activities of daily living. Patients are asked to respond to 13 questions | Responses measured on a 5-point Likert scale, with 0 indicating ‘not at all’ and 4 indicating ‘very much’, so the total score ranges from 0 to 52; the higher the score, the better the quality of life | 4-point increase from baseline | ≥40 |
| 36-Item Short Form Health Survey [4, 5] | General measure of patient health-related quality of life assessing the physical and mental components of health status as well as overall well-being  Consists of 36 questions belonging to eight domains:   - Physical well-being: four domains – physical functioning (ten items), role physical (four items), bodily pain (two items), and general health perceptions (five items) - Mental well-being: four domains – vitality (four items), social functioning (two items), role emotional (three items), and mental health (five items)   The physical well-being scores were aggregated to comprise the PCS and the mental well-being scores were aggregated to comprise the MCS | Scores were rescaled from 0 to 100 (converting the lowest possible score to 0 and the highest possible score to 100), with higher scores indicating a better quality of life | 2.5-point increase from baseline for physical component summary (PCS) and mental component summary (MCS) scores | ≥50 (converted score) |

**Table S2.** Baseline patient characteristics

|  | **Methotrexate (MTX) add-on study** | **Monotherapy study** |
| --- | --- | --- |
|  | Total population (N=594) | Total population (N=293) |
| **Patient demographics** | | |
| Age, mean (SE), years | 53.4 (0.51) | 52.1 (0.73) |
| Female, n (%) | 481 (81.0) | 231 (81.6) |
| **Disease characteristics** | | |
| Duration of RA, mean (SE), years | 8.3 (0.30) | 8.8 (0.43) |
| CRP, mean (SE), mg/L | 24.7 (1.04) | 27.2 (1.86) |
| TJC68, mean (SE) | 26.1 (0.57) | 26.1 (0.80) |
| SJC66, mean (SE) | 16.6 (0.37) | 16.8 (0.56) |
| DAS28 (CRP), mean (SE) | 6.1 (0.03) | 6.1 (0.05) |
| Anti-CCP positive, n (%) | 473 (79.8) | 225 (79.5) |
| RF positive, n (%) | 446 (75.2) | 211 (74.6) |
| Investigator’s Global Assessment, mean (SE) | 65.6 (0.63) | 69.6 (0.87) |
| **Prior treatments** | | |
| MTX duration, mean (SE), years | 5.0 (0.20) | 4.2 (0.27) |
| MTX dose, mean (SE), mg/week | 16.9 (0.18) | - |
| Corticosteroids, n (%) | 352 (59.3) | 189 (66.8) |
| bDMARD, n (%) | 50 (8.4) | 19 (6.5) |
| Antimalarials, n (%) | 53 (8.9) | 44 (15.5) |

Anti-CCP, anti–cyclic citrullinated peptide antibody; bDMARD; biologic disease-modifying antirheumatic drug; CRP, C-reactive protein; DAS28, disease activity score based on 28 joints; MTX, methotrexate; RA, rheumatoid arthritis; RF, rheumatoid factor; SE, standard error; SJC66, swollen joint count based on 66 joints; TJC68, tender joint count based on 68 joints

**Table S3**. Proportion of patients who achieved normative scores at Week 12 in the methotrexate add-on and monotherapy studies

| **Threshold for normative scores** | **Methotrexate add-on study dosing group (%)** | | | | | | | **Monotherapy study dosing group (%)** | | | |
| --- | --- | --- | --- | --- | --- | --- | --- | --- | --- | --- | --- |
|  |  | Once daily (q.d.) | | | Twice daily (b.i.d.) | | | Once daily (q.d.) | | | |
|  | Placebo | 50 | 100 | 200 | 25 | 50 | 100 | PBO | 50 | 100 | 200 |
| **Health Assessment Questionnaire-Disability Index (HAQ-DI) ≤0.5** | 10.5 | 23.2 | 27.1 | 30.2 | 23.3 | 22.4 | 33.3 | 5.6 | 16.7 | 22.9 | 27.5 |
| **Functional Assessment of Chronic Illness Therapy (FACIT)-Fatigue scale ≥40** | 23.3 | 36.6 | 45.9 | 43.0 | 39.5 | 35.3 | 42.9 | 16.7 | 33.3 | 37.1 | 40.6 |
| **Short Form-36: Mental component score ≥50** | 38.4 | 36.6 | 50.6 | 51.2 | 50.0 | 44.7 | 41.7 | 29.2 | 44.4 | 47.1 | 50.7 |
| **Short Form-36: Physical component score ≥50** | 3.5 | 13.4 | 9.4 | 11.6 | 14.0 | 8.2 | 22.6 | 4.2 | 6.9 | 12.9 | 13.0 |

**Figure Legends**

**Supplementary Figure 1.** Patient disposition during the methotrexate (MTX) add-on study (A) and the monotherapy study (B)

At Week 12, patients receiving placebo in the MTX add-on study and patients receiving placebo and filgotinib 50 mg q.d. in the monotherapy study who had not achieved a 20% improvement in swollen joint count and tender joint count were reassigned to receive filgotinib 100 mg q.d. (both studies) or 50 mg b.i.d. (MTX add-on study only).

**Supplementary Figure 2.** Proportion of subjects achieving normative HAQ-DI score (≤0.5) at Week 12 according to ACR20 responder status in the methotrexate (MTX) add-on study (A) and monotherapy study (B)

b.i.d., twice daily; HAQ-DI, health assessment questionnaire-disability index q.d., once daily.

**Supplementary Figure 3.** Proportion of subjects achieving normative FACIT-Fatigue score (≥40) at Week 12 according to ACR20 responder status in the MTX add-on study (A) and monotherapy study (B)

b.i.d., twice daily; FACIT, Functional Assessment of Chronic Illness Therapy; MTX, methotrexate; q.d., once daily

**Supplementary Figure 4.** Proportion of subjects achieving normative SF-36 PCS and MCS scores (≥50) at Week 12 according to ACR20 responder status. (A) PCS, MTX add-on, (B) PCS, monotherapy (C) MCS, MTX add-on, (D) MCS, monotherapy

b.i.d., twice daily; MCS, mental component score; MTX, methotrexate; PCS, SF-36 physical component score; q.d., once daily

1. Fries JF, Spitz P, Kraines RG, Holman HR. Measurement of patient outcome in arthritis. Arthritis Rheum 1980;23:137-45.

2. David Cella Ph.D, inventor FACIT-Fatigue questionnaire Version 4.

3. Cella D, Yount S, Sorensen M, Chartash E, Sengupta N, Grober J. Validation of the Functional Assessment of Chronic Illness Therapy Fatigue Scale relative to other instrumentation in patients with rheumatoid arthritis. J Rheumatol 2005;32:811-9.

4. Ware JE, Jr., Sherbourne CD. The MOS 36-item short-form health survey (SF-36). I. Conceptual framework and item selection. Med Care 1992;30:473-83.

5. McHorney CA, Ware JE, Jr., Raczek AE. The MOS 36-Item Short-Form Health Survey (SF-36): II. Psychometric and clinical tests of validity in measuring physical and mental health constructs. Med Care 1993;31:247-63.
